# Non-Conventional Methodologies in the Synthesis of 1-Indanones

**DOI:** 10.3390/molecules19055599

**Published:** 2014-04-30

**Authors:** Manuela Oliverio, Monica Nardi, Paola Costanzo, Luca Cariati, Giancarlo Cravotto, Salvatore Vincenzo Giofrè, Antonio Procopio

**Affiliations:** 1 Dipartimento di Scienze della Salute, Università Magna Graecia, Viale Europa, Germaneto (CZ) 88100, Italy; E-Mails: m.oliverio@unicz.it (M.O.); pcostanzo@unicz.it (P.C.); lucacariati@hotmail.it (L.C.); 2 Dipartimento di Chimica, Università della Calabria, Cubo 12C, Arcavacata di Rende (CS) 87036, Italy; E-Mail: monica.nardi@unical.it; 3 Dipartimento di Scienza e Tecnologia del Farmaco, Università di Torino, Via Pietro Giuria 9, Torino 10125, Italy; E-Mail: giancarlo.cravotto@unito.it; 4 Dipartimento Scienza del Farmaco e Prodotti per la salute, Università di Messina, Via Santissima Annunziata, Messina 98168, Italy, E-Mail: sgiofre@unime.it

**Keywords:** 1-indanones, microwaves, ultrasound, Q-tube™; Friedel-Crafts acylation

## Abstract

1-Indanones have been successfully prepared by means of three different non-conventional techniques, namely microwaves, high-intensity ultrasound and a Q-tube™ reactor. A library of differently substituted 1-indanones has been prepared via one-pot intramolecular Friedel-Crafts acylation and their efficiency and “greenness” have been compared.

## 1. Introduction

In the last two decades, environmental issues linked to the chemical and associated industries, such as the pharmaceutical industry, have become increasingly pertinent. Many classical synthetic methodologies require large amounts of natural resources and generate copious amounts of waste [1]. Thus, the need to incorporate green chemistry into the synthesis of active pharmaceutical ingredients and intermediates is of primary importance for the pharmaceutical industry [2].

Indanones and related compounds are important bioactive molecules. These compounds have exhibited biological activity against cancer cells and Alzheimer’s disease; moreover they can be used as synthetic intermediates for several drugs and as precursors to natural products [3,4,5,6,7,8,9,10]. Other applications include their use as ligands in olefinic polymerization catalysts [11,12,13,14,15,16,17,18,19,20] and as discotic liquid crystals [21]. The most famous drug which bears an indanone moiety is probably donepezil hydrochloride, which has been approved by the United States Food and Drug Administration (US-FDA) for the treatment of mild-moderate Alzheimer’s disease [22]. The intramolecular Friedel-Crafts cyclization reaction of 3-arylpropionic acids or chlorides is one of the most common methods for the preparation of 1-indanones [23,24,25,26,27,28,29]. Although the direct dehydrative cyclization of 3-arylpropionic acids is more difficult than cyclization via acid chlorides, it is preferable because of the environmental benefits it provides. In fact, the “one-step reaction” produces water as the only by-product while the “two-step reaction” generates a large amount of toxic and corrosive compounds. Nevertheless, direct cyclization usually requires an excess of protic acids (even as solvents) such as sulfuric acid, hydrogen fluoride [30], polyphosphoric acid [31], methanesulfonic acid (MSA) [32], a mixture of MSA and P_2_O_5_ [33], or Lewis acids such as AlCl_3_ and SnCl_4_ [30]. Some lanthanide triflates, in particular Tb(OTf)_3_, were reported to be useful catalysts for the dehydrative cyclization of 3-arylpropionic acids to form 1-indanones [34] in *o*-chlorobenzene at very high temperatures. We report herein an improved method for the synthesis of 1-indanone derivatives which proceeds via the superacid-catalyzed intramolecular Friedel-Crafts acylation of 3-arylpropionic acids.

As mentioned above, the examples of direct intramolecular Friedel-Crafts acylation of 3-arylpropionic acids to produce 1-indanone derivatives are very far from satisfying many green chemistry principles since they are performed under extremely drastic experimental conditions, such as elevated reaction temperatures (250 °C in chlorobenzene) and require very long reaction times [23,24,25,26,27,28,29,30,31,32,33,34]. In light of the enormous diffusion and application of non-conventional techniques in the implementation of synthetic green processes [35,36,37,38], and our own experience in this field [39,40,41,42,43,44], we decided to explore the use of microwave irradiation (MW), high-intensity ultrasound (US) and high-pressure conditions to develop a greener synthesis of 1-indanone derivatives via the intramolecular Friedel-Crafts acylation of 3-arylpropionic acids.

## 2. Results and Discussion

**Scheme 1:**
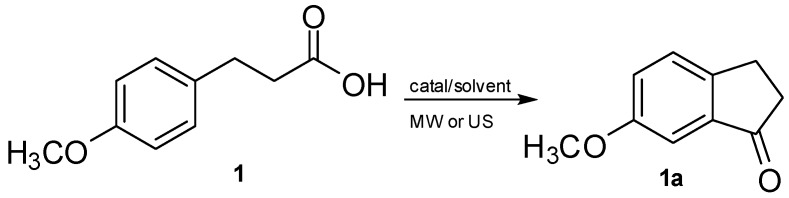
Synthesis of 1-indanone **1a** via the cyclization of 3-(4-methoxyphenyl) propionic acid **1** under US or MW irradiation.

To start our investigation, it was decided to study the cyclization of 3-(4-methoxyphenyl)propionic acid (**1**) in different solvents and experimental conditions using Tb(OTf)_3_ as the Lewis acid, the best reported in the literature [34], and triflic acid [27] (Scheme 1). All attempts to perform the MW-assisted Tb(OTf)_3_ catalyzed reaction in environmentally benign solvents such as PEG, *n*-butanol, ethyl lactate or water, or even in slightly activated aromatic solvents failed (Table 1, entries 1–6).

A negative result was also observed when the reaction was performed in inert high boiling solvents such as isooctane (Table 1, entry 7) and only in chlorobenzene a good conversion of substrate was observed after only 60 min, albeit affording a low yield of product **1a** (Table 1, entry 8).

**Table 1:** MW _*vs.*_ US in the intramolecular Friedel-Craft acylation of 3-(4-methoxyphenyl) propionic acid (**1**).

Entry	Catalyst (%mol)	Solvent	T (°C)	Method	Time (min)	Conv. (%)	Yield (%)
1	Tb(OTf)_3_ (20)	PEG ^a^	180	MW	30	-	-
2	Tb(OTf)_3_ (20)	n-BuOH ^a^	180	MW	30	-	-
3	Tb(OTf)_3_ (20)	Ethyl lactate ^a^	180	MW	30	-	-
4	Tb(OTf)_3_ (20)	H_2_O	180	MW	30	-	-
5	Tb(OTf)_3_ (20)	Toluene ^b^	180	MW ^c^	30	10	10
6	Tb(OTf)_3_ (20)	Xylene ^b^	180	MW ^c^	30	-	-
7	Tb(OTf)_3_ (20)	Isooctane ^c^	250	MW ^c^	10	-	-
8	Tb(OTf)_3_ (20)	Cl-benzene	250	MW	60	85	33 ^d^
9	TfOH (10 eq.)	CH_2_Cl_2_ (dry)	25	r.t.	1440	85	61 ^d^
10	TfOH (3 eq.)	CH_2_Cl_2_ (dry)	80	MW	60	100	100
11	TfOH (1 eq.)	CH_2_Cl_2_ (dry)	80	MW	120	50	20 ^d^
12	TfOH (2 eq.)	CH_2_Cl_2_ (dry)	80	MW	60	75	53 ^d^
13	TfOH (3 eq.)	CH_2_Cl_2_ (dry)	110	MW	30	100	100
14	TfOH (3 eq.)	CH_2_Cl_2_ (dry)	40	US	120	-	-
15	TfOH (5 eq.)	CH_2_Cl_2_ (dry)	40	US	1260	80	80
16	TfOH (10 eq.)	CH_2_Cl_2_ (dry)	40	US	150	100	100
17	TfOH-SiO_2_ (30)	CH_2_Cl_2_ (dry)	110	MW	60	-	-
18	TfOH-SiO_2_ (30)	CH_2_Cl_2_ (dry)	40	US	60	-	-

^a^ Formation of side-esterification or trans-esterification by-products; ^b^ Formation of intermolecular Friedel-Craft acylation by-products; ^c^ Reaction conducted in the presence of a SiC tablet used as a MW inert absorbent; ^d^ Formation of by-products.

Clearly improved results were obtained when the reaction was catalyzed by a large excess of triflic acid (TfOH) in CH_2_Cl_2_. As previously reported in the literature [27], a 61% yield of **1a** was observed at room temperature, but only after a very long reaction time (Table 1, entry 9), whereas complete conversion of substrate **1** to indanone **1a** was observed after only 60 min when the reaction was performed under MW at 80 °C using three equivalents of TfOH (Table 1, entry 10).

Lower amounts of catalyst gave poorer yields due to the considerable number of by-products formed (Table 1, entries 11 and 12). The same positive result was registered in only 30 min when the reaction temperature was raised to 110 °C (Table 1, entry 13). Comparable results were registered when the same reaction was accomplished under US, however, the complete conversion of the substrate **1** to indanone **1a** was observed in an acceptable reaction time only when a large excess of triflic acid was used in CH_2_Cl_2_ at 40 °C (Table 1, entries 14–16). Triflic acid is a highly corrosive and fuming liquid. It is the strongest Brønsted acid, having a H_o_ value of −14.1. Unmodified chromatographic silica gel-supported triflic acid has very recently been proposed as an efficient and recyclable catalyst under solvent free conditions [45]. Thus, in an attempt to add this more environmentally friendly way to use this superacid to our synthetic protocol, the test reaction reported in Scheme 1 was also performed using silica gel supported triflic acid (TfOH-SiO_2_). Unfortunately, no conversion (of substrate **1**) was observed after 60 min under MW or US (Table 1, entries 17 and 18). In a last test, the MW- and US-assisted reactions were performed in significantly lower reaction times in dry CH_2_Cl_2_, to give quantitative yields of product **1a** and avoiding the formation of by-products. All the attempts to use more environmentally acceptable solvents gave scarce results, leaving dry CH_2_Cl_2_ as the best choice for performing the reaction reported in Scheme 1.

Performing the reaction in dry CH_2_Cl_2_ permitted the total conversion of the substrate **1** into **1a** in 60 min at 80 °C using only 3 equivalents of TfOH (Table 1 entry 10). On the other hand, in the US-assisted protocol, the same reaction was performed at lower temperature with a cleaner reaction profile and simplified work-up, despite the significantly higher amount of catalyst necessary to obtain the quantitative conversion of **1** into **1a**.

In the attempt to unify the advantages of both assisted methods, the reaction pictured in Scheme 1 was performed in the Q-tube™ which is a safe pressure reactor which features a patent pending pressure-release and reseal system that prevents accidental explosions due to over–pressurization. Q-tube™ is an affordable alternative to expensive and cumbersome MW synthesizers; this system enables a reaction to be carried out at higher temperature than the boiling point of solvents and reagents, which will increase the reaction rate.

The results reported in Table 2, clearly confirm the efficiency of Q-tube™ as a valid alternative technique (Table 2, entry 1), which provides a cleaner reaction profile very similar to what is observed in the US-assisted protocol. Longer reaction times did not improve the reaction performance and only traces of product **1a** were obtained at 150 °C (Table 2, entries 2 and 3). It is of interest to know if Tb(OTf)_3_, the most active Lewis acid reported in literature for this process, can be used as catalyst in the Q-tube experiments. A poor result was registered when the reaction was carried out in chlorobenzene as reported [34] (Table 2, entry 4), whereas a good yield of product **1a** was obtained when the reaction was performed in toluene (Table 2, entry 5).

**Scheme 2:**
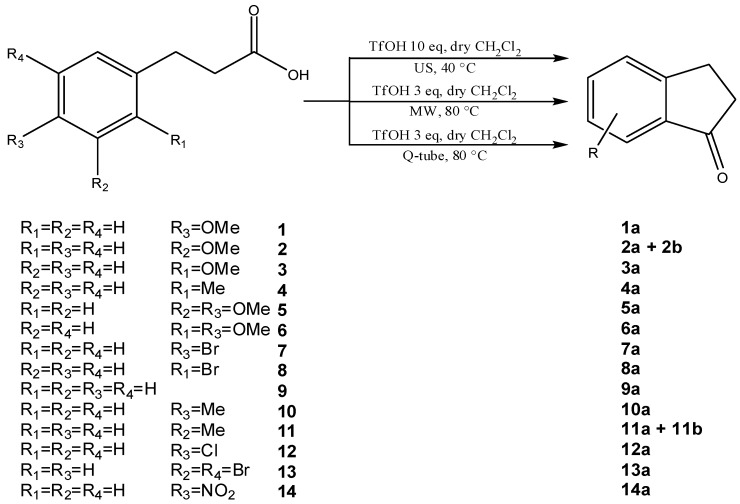
MW-, US-assisted and Q-tube™ protocols for 1-indanone synthesis applied to several aryl-3-propionic acids.

Very poor results were obtained when the reaction was performed in high-boiling point hydrocarbon solvents and at very high reaction temperatures (Table 2, entries 7 and 8). Finally, the endeavor to carry out the reaction again using silica gel-supported triflic acid in CH_2_Cl_2_ gave a negative result (Table 2, entries 9 and 10) leaving the following three experimental procedures as the best choices for the reaction depicted in Scheme 1; triflic acid is added at 0 °C to a solution of 0.5 mmol of substrate in dry CH_2_Cl_2_, then the solution is heated to room temperature and the reaction is conducted under MW- or US-assistance or in the Q-tube™ (Table 1 entries 10, 16 and Table 2 entry 1 respectively). The best experimental conditions obtained with MW, US- and Q-tubeTM protocols were applied to several substrates (Scheme 2) with the aim of testing the applicability of the methods and comparing their efficiency and versatility (Table 3).

**Table 2:** Q-Tube method in the intramolecular Friedel-Craft acylation of 3-(4-methoxyphenyl) propionic acid **1**.

Entry	Catalyst (%mol)	Solvent	T (°C)	Time (min)	Conv. (%)	Yield (%) ^a^
1	TfOH (3 eq.)	CH_2_Cl_2_ (dry)	80	60	100	100
2	TfOH (3 eq.)	CH_2_Cl_2_(dry)	110	30	100	96
3	TfOH (3 eq.)	CH_2_Cl_2_(dry)	150	10	100	trace ^b^
4	Tb(OTf)_3_ (10)	C_6_H_5_Cl	180	180	100	40
5	Tb(OTf)_3_ (10)	toluene	150	180	100	86 ^c^
6	Tb(OTf)_3_ (10)	n-C_7_H_14_	180	240	-	-
7	Tb(OTf)_3_ (20)	n-C_7_H_14_	250	120	18	45
8	Tb(OTf)_3_ (10)	isooctane	250	240	32	20
9	TfOH-SiO_2_(30 )	CH_2_Cl_2_ (dry)	25	120	-	-
10	TfOH-SiO_2_ (30)	CH_2_Cl_2_ (dry)	180	180	-	-

^a^ Isolated yields; ^b^ The high temperature broke the Teflon septum; ^c^ Formation of intermolecular Friedel-craft acylation by-products.

**Figure 1:**
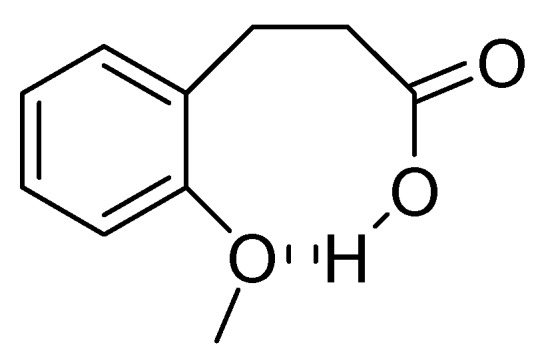
Proposed unfavourable electronic effect involved in the failure of the 3-(2-methylphenyl)propionic acid **3** intramolecular cyclization.

In the case of 3-(2-methoxyphenyl)propionic acid (**3**), the only available cyclization site is the non-activated 6-position on the aromatic ring, so that no product formation was observed and only polymerized products were detected (Table 3, entry 3). Moreover, an unfavorable electronic effect can be invoked to explain the failure of the intramolecular cyclization in substrate **3**. As depicted in Figure 1, an H-bond can constrain the acidic OH to form a transient stable ring and keep the carboxylic moiety distant from the aromatic ring. This hypothesis is confirmed by the results observed for 3-(2-methylphenyl)propionic acid where the absence of the ether oxygen prevents the formation of the transient ring through the O-H interaction leaving the carboxylic group free to attack the non-activated 6-position on the aromatic ring and furnishing a quantitative yield of the 1-indanone derivative **4a** (Table 3, entry 4).

**Table 3:** MW _*vs.*_ US method in the intramolecular Friedel-Craft acylation of phenyl propionic acids **1**–**7**.

Entry	Product	US-Assisted Reaction ^a^			MW-Assisted Reaction ^a^			Q-Tube-Assisted Reaction ^a^		
		Time (min)	Conv (%)	Yield (%)	Time (min)	Conv (%)	Yield(%)	Time (min)	Conv (%)	Yield(%)
1	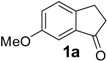	150	100	100	60	100	100	60	100	100
		210 ^b^	100	100	90 ^b^	100	100	90 ^b^	100	100
2	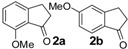	60	100	88/12	60	100	90/10	60	100	90/10
				(**2b**)/(**2a**)			(**2b**)/(**2a**)			(**2b**)/(**2a**)
3	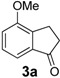	120	100	- ^c^	180	100	-^ c^	180	100	-^ c^
4	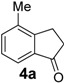	60	100	100	60	100	100	60	100	100
5	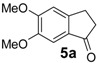	60	100	100	60	100	100	60	100	100
6	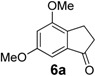	360	-	-	180	-	-	180	-	-
7	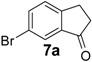	360	100	100	180 ^d^	42	33	180 ^d^	54	36
					60 ^e^	100	100	60 ^e^	100	100
8	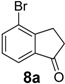	1200	90	90	180 ^d^	58	48	180 ^d^	44	43
					60 ^e^	100	100	60 ^e^	100	100
9	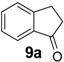	60	100	100	180	100	100	180	100	100
10	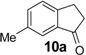	60	100	100	60	100	100	60	100	100
11	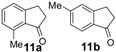	60	100	85/15	60	100	88/12	60	100	88/12
				(**11b**)/(**11a**)			(**11b**)/(**11a**)			(**11b**)/(**11a**)
12	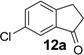	400	100	100	180 ^d^	38	30	180 ^d^	45	33
					60 ^e^	100	100	60 ^e^	100	100
13	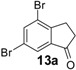	460	100	100	180 ^d^	32	28	180 ^d^	40	36
					120 ^e^	100	100	120 ^e^	100	100
14	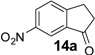	1200 ^d^	-	-	360 ^e^	-	-	360 ^e^	-	-

^a^ All new products were characterized by GC/MS and ^1^H-NMR spectroscopy; all the known products were characterized by comparison with the spectral data in the literature; ^b^ Reaction conducted with 5.0 mmol of reactant; ^c^ Formation of poli-ketones by polymerization; ^d^ Reaction conducted in presence of 5 eq. of TfOH; ^e^ Reaction conducted in presence of 10 equivalents of TfOH.

Similarly, the unfavourable electronic effect associated with greater steric hindrance can explain the extremely poor reactivity in the case of 3-(2,4-dimethoxyphenyl) propionic acid (**6**) where only the starting material was recovered in all three reaction systems, even after prolonged reaction times (Table 3, entry 6). In all the other reported examples quantitative product yields were observed for all three activation methodologies in very short reaction times and without significant by-product formation (Table 3, entries 1, 2, 5, 8–13). It is worth noting that the dis-activated halo-substituted phenylpropionic acids **7**, **8**, **12** and **13** gave quantitative yields for the direct intramolecular cyclization which, to the best of our knowledge, has never reported before. However, no conversion was observed in the case of the highly deactivated 3-(4-nitrophenyl)propionic acid (**14**) under all the experimental conditions adopted (Table 3, entry 14), even after very prolonged reaction times and using higher amounts of catalyst.

Finally, in order to test the scalability of the protocol, a scaled-up reaction was realized on 5.0 mmol of 3-(4-methoxyphenyl)propionic acid (**1**) (Table 3, entry 1, line 2), giving rise to the same results in terms of conversion, yield and reaction profile, but after a slightly longer reaction time. All the synthetic methodologies tested in this work enable much shorter reaction times and use of an acceptable amount of triflic acid [27]. *o*-Chlorobenzene was replaced by CH_2_Cl_2_ [34]. The comparison of the three techniques reported in Table 3 shows that MW-assisted reactions could be performed using only 3 equivalents of triflic acid in shorter reaction times than the other protocols. On the other hand, US-assistance allows the reaction to be conducted at lower temperature and with a significantly cleaner profile, simplifying the reaction work-up despite the higher amount of triflic acid required. The Q-tube™ technology gave the same results as the MW-assisted protocol but with cleaner reaction profiles, offering a good alternative to MW or US technologies in terms of yield, safety and efficiency.

## 3. Experimental

### 3.1. General Information

All chemicals were obtained from Sigma-Aldrich or Acros Organics (Geel, Belgium) and used as received. All solvents were distilled using standard methods before use. All reactions were carried out in flame-dried glassware, under a dry nitrogen atmosphere. MW-assisted reactions were performed on a Synthos 3000 instrument from Anton Paar (Torino, Italy), equipped with a 64MG5 Rotor and an IR probe used for external temperature control. US-assisted reactions were performed in a high-power US-bath (19.6 kHz) made by Danacamerini (Torino, Italy). Q-Tube assisted reactions were performed in a Q-tube™ safe pressure reactor from Q Labtech (distributed by Sigma-Aldrich), equipped with a cap/sleeve, a pressure adapter (120 psi), a needle adapter/needle, a borosilicate glass tube, a Teflon septum and a catch bottle. Reactions were monitored using a GC–MS Thermo Fisher Scientific workstation, composed of a Focus GC (Thermo TR, Waltham, MA, USA- 5 ms SQC 15 m × 0.25 mm ID × 0.25 µm, working on split mode, 1.2 mL/min using He as the carrier gas) and a DSQ II mass detector. TLC were performed using Kielsegel 60-F264 on aluminium plates, commercially available from Merck (Darmstadt, Germany). Liquid flash chromatography was performed on a Supelco VERSA FLASH HTFP station (distributed by Sigma-Aldrich) using silica cartridges commercially available from Supelco. ^1^H-NMR spectra were recorded on a Bruker WM 300 instrument (Milano, Italy) on samples dissolved in CDCl_3_. Chemical shifts are given in parts per million (ppm) from tetramethylsilane as the internal standard (0.0 ppm). All products in this report are known and were characterized by standard techniques (^1^H- and ^13^C-NMR, GC/MS) and the data were compared with those reported in the literature [33,46,47,48] for identification.

### 3.2. General US-Assisted Procedure

Trifluoromethane sulfonic acid (10 eq.) was gently added to a cooled (0 °C) solution of a 3-phenyl propionic acid (0.5 mmol) in dry CH_2_Cl_2_ (0.5 mL) in a two-necked round bottom flask. The temperature was raised to room temperature. The mixture was reacted in a high-power US-bath (19.6 kHz) at 40 °C. The reaction was monitored by TLC and GC/MS until the reactant disappeared. The mixture was poured into ice and extracted three times with CH_2_Cl_2_. The organic phase collected was dried over Na_2_SO_4_, filtered and concentrated under vacuum. The desired pure product was separated from the crude by flash chromatography.

### 3.3. General MW-Assisted Procedure

Trifluoromethanesulfonic acid (3 eq.) was gently added to a cooled (0 °C) solution of a 3-phenyl propionic acid (0.5 mmol) in dry CH_2_Cl_2_ (1.0 mL) in 3 mL glass vial using a Synthos 3000 microwave oven (Anton-Paar). The temperature was raised to room temperature. Appropriate Teflon and screw caps were placed on the top of the vial. The mixture was heated in the MW reactor at 80 °C in “power-controlled mode” for the appropriate time. The reaction was monitored by TLC and GC/MS until the reactant disappeared. The mixture was poured onto ice and extracted three times with CH_2_Cl_2_. The collected organic phase was dried on Na_2_SO_4_, filtered and concentrated under vacuum. The desired pure product was separated from the crude by flash chromatography.

### 3.4. General Q-tube^tm^-Assisted Procedure

Trifluoromethane sulfonic acid (3 eq.) was gently added to a cooled (0 °C) solution of a 3-Phenyl propionic acid (0.5 mmol) in dry CH_2_Cl_2_ (1.0 mL) in a 12 mL Q-tube™ pressure tube, furnished by Q Labtech. The temperature was raised to room temperature. A Teflon septum was placed on the top of the tube and the appropriate cap and pressure adapter were used. The mixture was heated in an oil bath at 80 °C. The reaction was monitored by TLC and GC/MS until the reactant disappeared. The mixture was poured into ice and extracted three times with CH_2_Cl_2_. The organic phase collected was dried on Na_2_SO_4_, filtered and concentrated under vacuum. The desired pure product was separated from the crude by flash chromatography.

## 4. Conclusions

We have reported the application of three different non-conventional techniques to the synthesis of a library of substituted 1-indanones, via the direct Friedel-Crafts intramolecular cyclization of arylpropionic acids. The comparison between three alternative reaction methodologies, namely MW, US and Q-Tube™ assisted reactions, showed that Q-tube™ equipment can be proposed as a valid alternative to monomode MW and US technologies in term of efficiency, safety (virtually eliminating the risk of pressure explosions) and a cleaner reaction profile.

## Supplementary Materials

Supplementary materials can be accessed at: http://www.mdpi.com/1420-3049/19/5/5599/s1.
