# Supplementary material for: Non-Conventional Methodologies in the Synthesis of 1-Indanones
Source: Molecules. 2014 Apr 30;19(5):5599–610. doi: 10.3390/molecules19055599 (PMC6271961; doi:10.3390/molecules19055599)
Supplement: Supplementary file 1 [file molecules-19-05599-s001.pdf]

Supplementary Material

^{1}H- and ^{13}C-NMR Spectra of 1a

6-Methoxy-2,3-dihydro-1H-inden-1-one (**1a**)

^{1}H-NMR δ (ppm) (300 MHz, CDCl3): 2.6–2.8 (m, 2H$_1$), 3.0–3.1 (m, 2H$_2$), 3.8 (s, 3H, OMe), 7.1–7.2 (m, 1H$_3$), 7.3–7.4 (m, 2H$_{4,5}$); ^{13}C-NMR (300 MHz, CDCl$_3$): δ 205, 160, 148, 139, 126, 125, 105, 55, 37, 25.

Figure S1. ^{1}H- and ^{13}C-NMR Spectra of 6-Methoxy-2,3-dihydro-1H-inden-1-one (**1a**).

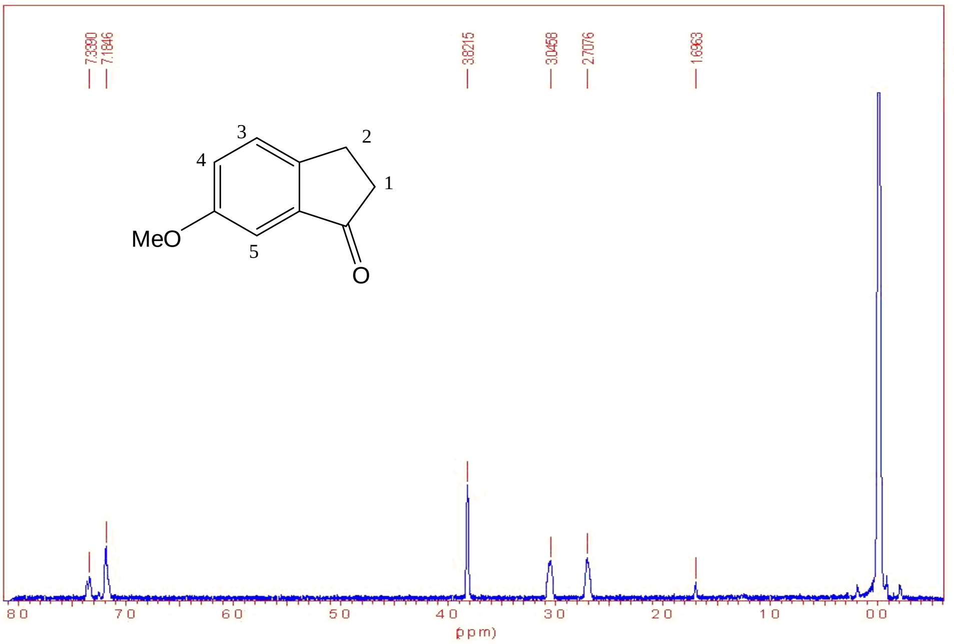

^{1}H-NMR

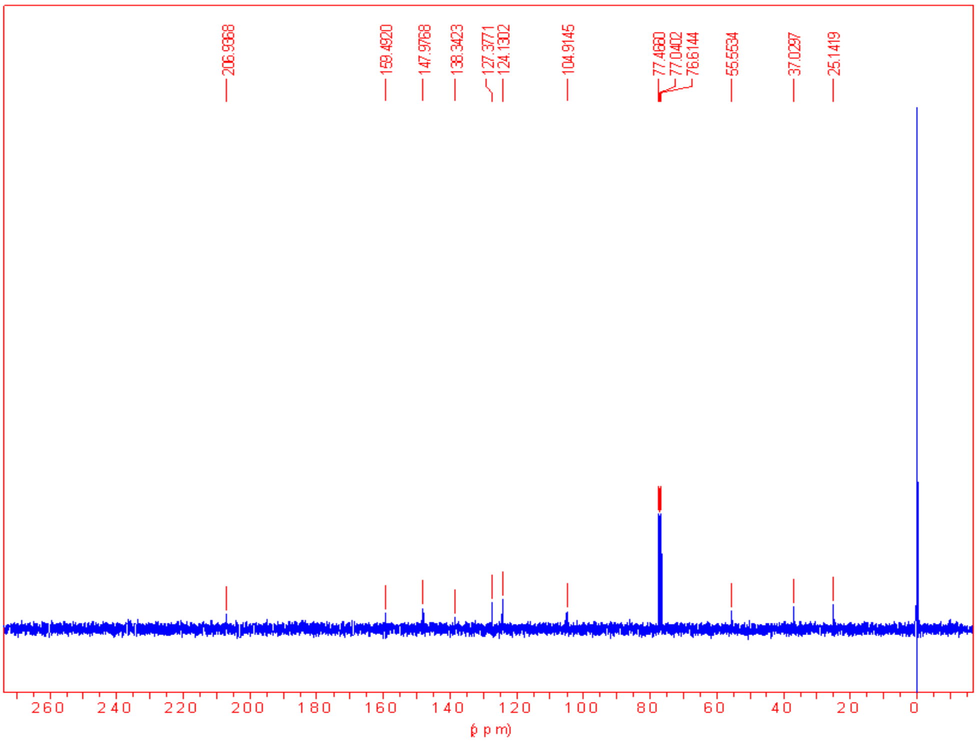

^{13}C-NMR

^{1}H- and ^{13}C-NMR Spectra of 4a

4-Methyl-2,3-dihydro-1H-inden-1-one (**4a**)

^{1}H-NMR δ (ppm) (300 MHz, CDCl3): 2.36 (s, 3H_3), 2.67–2.73 (m, 2H_1) 3.02 (t, 2H_2, *J* = 6Hz), 7.29 (t, 1H_5, *J* = 7Hz), 7.40 (d, 1H_4, *J* = 7Hz), 7.60 (d, 1H_6, *J* = 7Hz). ^{13}C-NMR (300 MHz, $CDCl_3$): δ 207.4, 154.1, 136.9, 135.9, 135.0, 127.5, 121.1, 36.2, 24.6, 17.7.

Figure S2. ^{1}H- and ^{13}C-NMR Spectra of 4-Methyl-2,3-dihydro-1H-inden-1-one (**4a**).

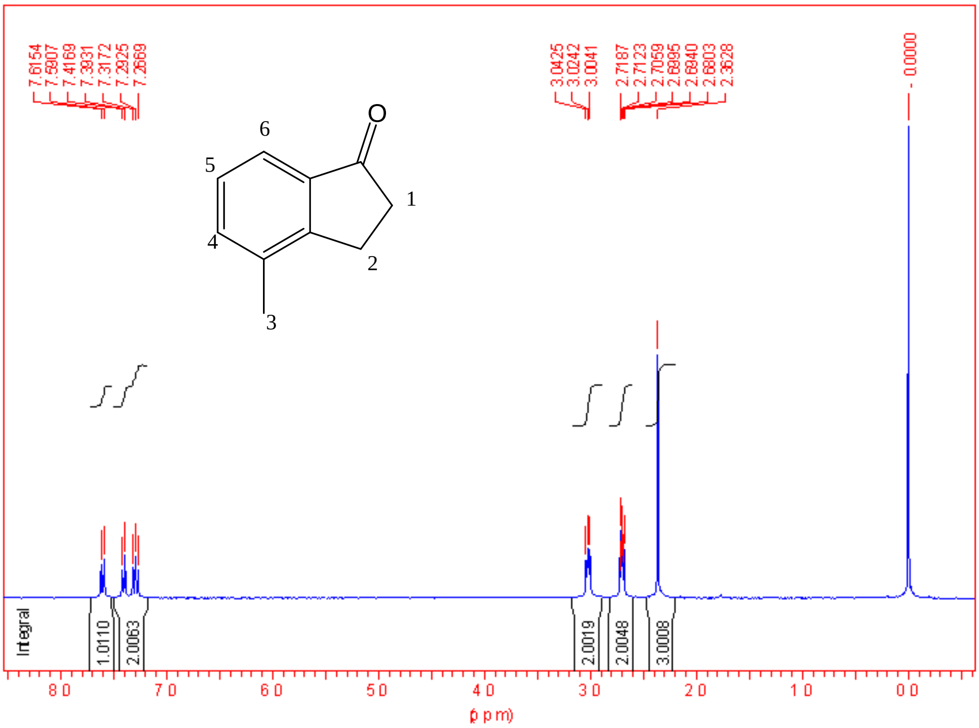

^{1}H-NMR

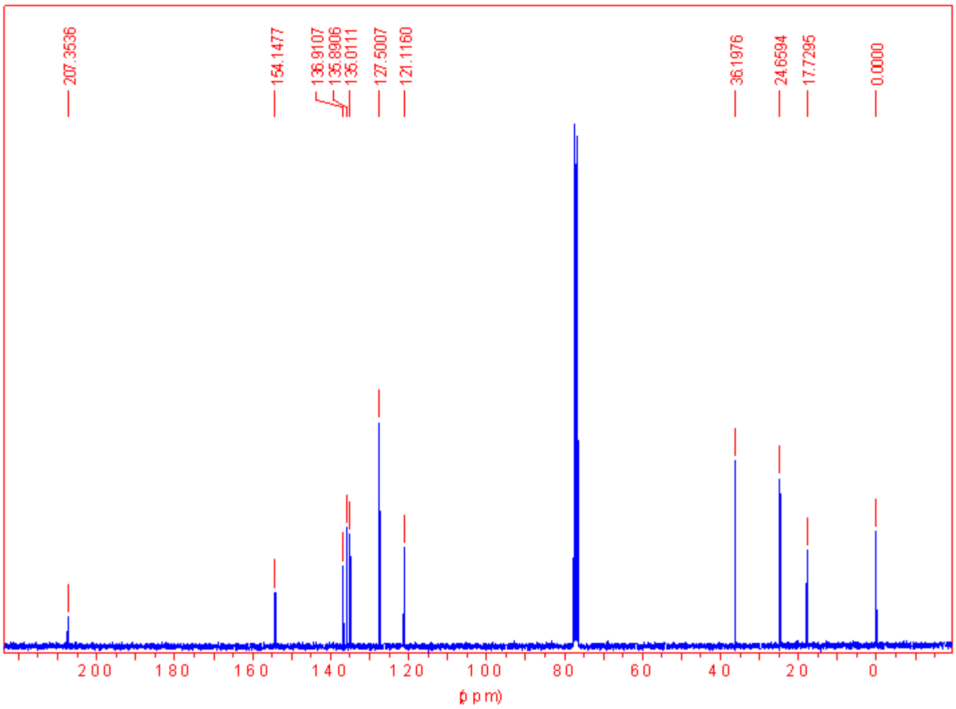

^{13}C-NMR

^{1}H- and ^{13}C-NMR spectra of 5a

5,6-dimethoxy-2,3-dihydro-1H-inden-1-one (**5a**)

^{1}H-NMR δ (ppm) (300 MHz, CDCl3): 2.68 (m, $2H_1$), 3.06 (t, $2H_2$, J = 6Hz), 3.91 (s, $3H_4$), 3.98 (s, $3H_5$), 6.90 (s, $1H_3$), 7.19 (s, $1H_{6}$). ^{13}C-NMR (300 MHz, $CDCl_3$): δ 205.7, 155.4, 150.4, 149.4, 129.9, 107.5, 104.2, 56.2, 56.1, 36.5, 25.6.

Figure S3. ^{1}H- and ^{13}C-NMR spectra of 5,6-dimethoxy-2,3-dihydro-1H-inden-1-one (**5a**).

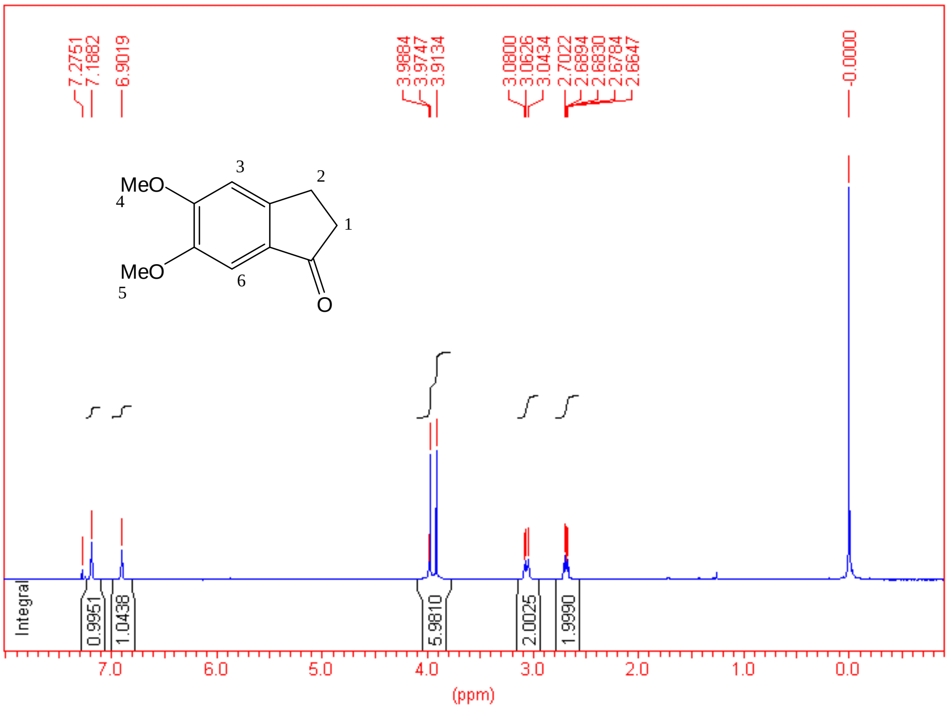

^{1}H-NMR

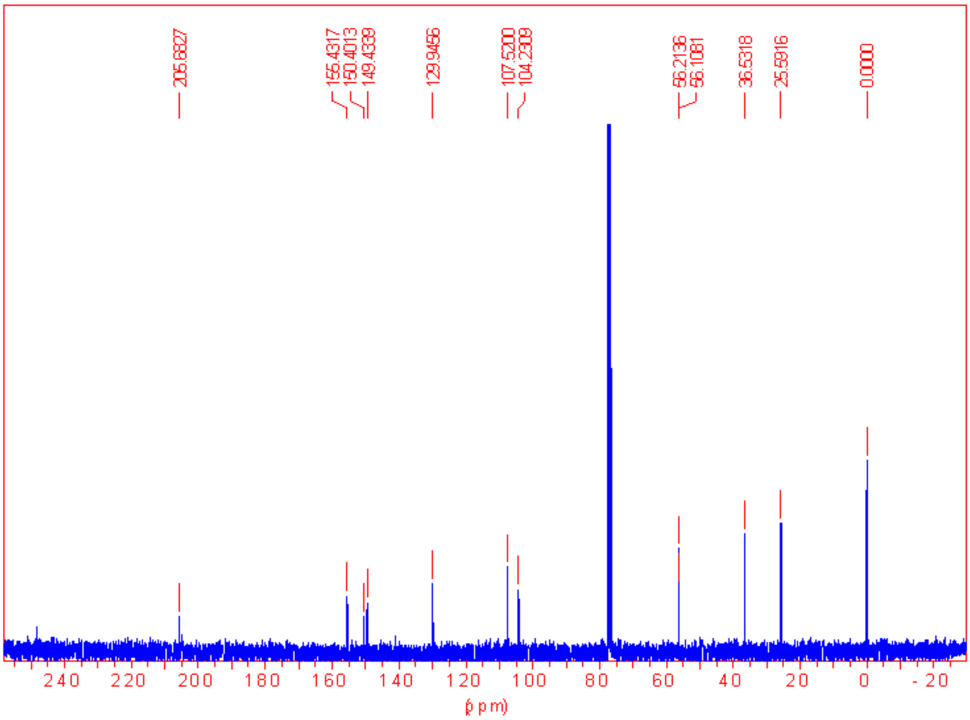

^{13}C-NMR

^{1}H- and ^{13}C-NMR Spectra of 9a

2,3-dihydro-1H-inden-1-one (**9a**)

^{1}H-NMR δ (ppm) (300 MHz, CDCl3): 2.69 (m, 2H$_1$), 3.15 (t, 2H$_2$, J = 6Hz), 7.37 (m, 1H$_5$); 7.48 (m, 1H$_3$), 7.60 (m, 1H$_4$), 7.76 (m, 1H$_6$). ^{13}C-NMR (300 MHz, CDCl$_3$): δ 207.1, 155.2, 137.1, 134.6, 127.3, 126.7, 123.7, 36.2, 25.8.

Figure S4. ^{1}H- and ^{13}C-NMR Spectra of 2,3-dihydro-1H-inden-1-one (**9a**).

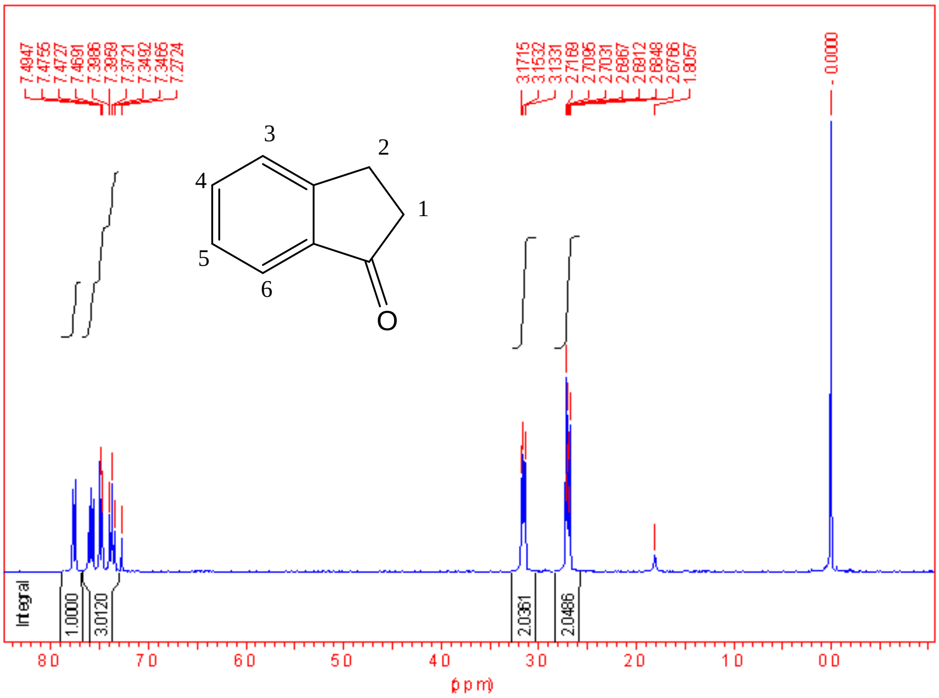

^{1}H-NMR

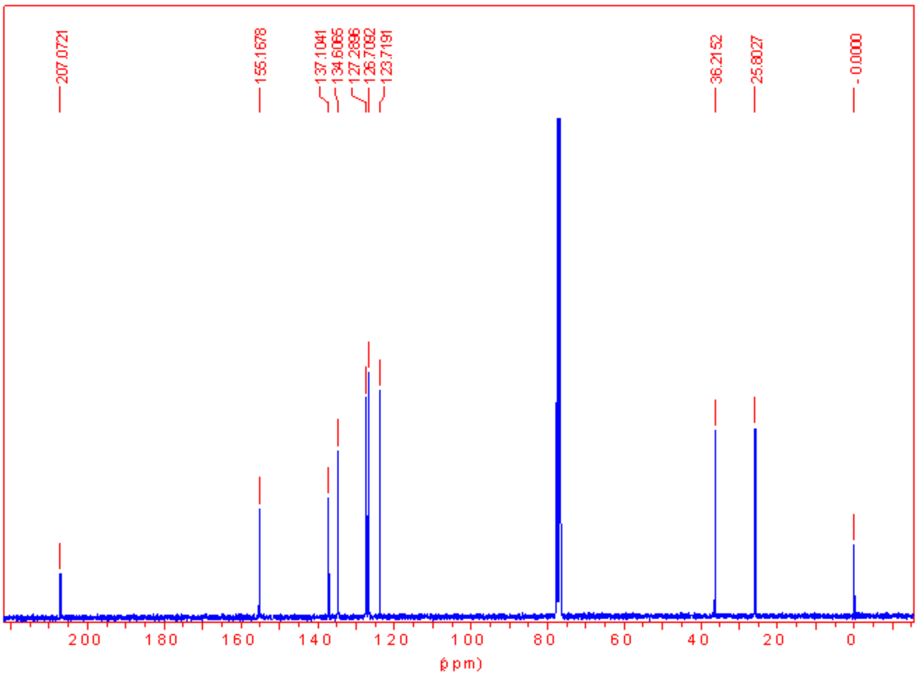

^{13}C-NMR
